# Dangerously Intelligent: A Call for Re-Evaluating Psychopathy Using Perceptions of Intelligence

**DOI:** 10.3390/jintelligence11020025

**Published:** 2023-01-23

**Authors:** Sergio A. Silverio, Minna T. Lyons, Sam P. Burton

**Affiliations:** 1 School of Life Course & Population Sciences, Faculty of Life Sciences & Medicine, King’s College London, London SE1 7EH, UK; 2 School of Psychology, Faculty of Health, Liverpool John Moores University, Liverpool L3 3AF, UK; 3 Department of Psychology, Institute of Population Health, University of Liverpool, Liverpool L69 7ZA, UK

**Keywords:** intelligence, psychopathology, individual differences, relationship cognition, opposite-sex judgements

## Abstract

Background: Primary psychopathy (i.e., unemotional and callous predisposition) is associated with career, educational, and general life success, whereas secondary psychopathy (i.e., impulsivity and risk-taking) relates to criminality, hedonistic lifestyles, and detrimental behaviours. Although psychopathy sub-types have differential relationships to career and life success, how these traits are perceived by others relating to intelligence has not previously been researched. It is also unclear what role an individual’s own psychopathy score plays in perceptions of intelligence. Methods: In an online experiment (n = 458), we investigated perceptions of intelligence (via a combined proxy of whether the rater thought the character in the vignettes had a high IQ and had attended university), using 12 vignettes of high and low primary and secondary psychopathic individuals. Results: High-secondary-psychopathy vignettes were perceived as least intelligent (in agreement with the literature which states people high in secondary psychopathy traits are usually involved in petty crimes, risk taking, and substance abuse, and therefore perceived as socially undesirable). Low-secondary-psychopathy vignettes were perceived significantly more intelligent than high-primary-psychopathy vignettes (in-line with the literature suggesting the placidity and kindness which comes with being low in psychopathic traits is an amenable quality in our society). There was evidence for assortative intelligence perceptions: those high-primary psychopathy self-scorers perceived primary psychopathy vignettes as intelligent (which could be evidence of a ‘likes attract’ phenomenon, whereby those high in primary psychopathy admire others who are similarly high in primary psychopathy). Conclusions: The results suggest individuals demonstrating risk-taking behaviours are perceived as least intelligent, supporting previous research associating secondary psychopathy with poor academic or career success.

## 1. Introduction

In recent years, there has been a surge of research investigating trait psychopathy and its relationship to life success in domains such as amassed wealth and increased income (Boccio and Beaver 2015; Ullrich et al. 2008; Wallace et al. 2022), and consistent, high attainment in academic study (Hassall et al. 2015; Baran and Jonason 2020). Likewise, psychopathy has been linked to career progression, promotion, and advancement (Akhtar et al. 2013; Howe et al. 2014; Lilienfeld et al. 2014; Spurk et al. 2015). Psychopathy is considered a personality disorder characterised by low empathy or remorse, strong traits of self-preservation and egotism, and disinhibition (Edens et al. 2006). The two-factor structure model of psychopathy has been widely studied in the personality literature, with research from Falkenbach et al. (2017) suggesting self-report measures can divide the construct into two facets: primary and secondary (see also Skeem et al. 2007). The findings of many studies indicate secondary psychopathy (i.e., impulsivity and risk-taking) relates to lower success (Hassall et al. 2015; Ullrich et al. 2008), whereas primary psychopathy (i.e., unemotional and callous predisposition) either lacks an association with success (Ullrich et al. 2008) or relates to higher success (Akhtar et al. 2013; Howe et al. 2014). Indeed, these findings partially support the idea that primary psychopathy is the successful facet of psychopathy, beneficial in settings such as the corporate world (Babiak and Hare 2006; Gao and Raine 2010). Secondary psychopathy, in turn, is associated with risk-taking (Lyons 2015), petty crimes (Lyons and Jonason 2015), and incarceration (Gao and Raine 2010), leading to less desirable outcomes in life. However, it is important to realise that these factors are, at times, overlapping and not always entirely mutually exclusive (Edens et al. 2006).

Success is not the only way in which people displaying primary and secondary psychopathic tendencies differ. For example, during social interactions, individuals high in primary (but not secondary) psychopathy tend to be charming, at ease holding court, and convincingly lie without trouble or concern. Furthermore, people high in primary psychopathy tend to dominate conversations (Manson et al. 2014). Dominant people are perceived as proficient, even when they lack actual competence (Anderson and Kilduff 2009), which could explain the career success of those high in primary psychopathy. Furthermore, there are differences between the two sub-types in the activity levels at different times of the day, whereby individuals high in primary psychopathy had a tendency towards a morning chronotype, whereas secondary psychopathy was associated with an evening chronotype (Jonason et al. 2013). Those who prefer early starts are perceived as better employees in the workplace (Yam et al. 2014); this could be one of the factors behind the career success of employees who are high in primary psychopathy. 

The success of individuals high in primary psychopathy could also be related to a third variable, namely intelligence, which relates to academic and career success (Strenze 2007) and less likelihood of redundancy from the workplace (Furnham and Petrides 2006). Different forms, such as social and cognitive intelligence (Aslam et al. 2016), emotional intelligence (de Haro Garcia and Castejón Costa 2014; Sultana et al. 2016), and crystallised intelligence (Hagmann-von Arx et al. 2016) have all been positively associated with career success. Interestingly, we seem to have the ability to assess the intelligence of strangers, even after a brief exposure to a videotape (Borkenau and Liebler 1993) or a still photograph (Kleisner et al. 2014), and our judgements about intelligence are based on multiple cues, even including voice-pitch (Schroeder and Epley 2015). Implicit intelligence judgements can have real-life consequences, for example, impacting employment decisions (Schroeder and Epley 2015) or with whom to go out on a date (Karbowski et al. 2016).

There currently is very little empirical evidence for perceived and actual intelligence in relation to primary and secondary psychopathy in normative samples. This is surprising given the links between career success and intelligence (Furnham and Petrides 2006), and the associations between primary psychopathy and career success (Spurk et al. 2015). It would therefore be expected that those who score high on primary psychopathy, compared with low-primary psychopathy individuals, are more intelligent, or are at least perceived as being intelligent. The existing evidence regarding the psychopathy-intelligence link is conflicting, and comes mainly from forensic and psychiatric samples, which makes it difficult to generalise to the rest of the population (see DeLisi et al. 2010; Demakis et al. 2015; de Tribolet-Hardy et al. 2014; Sharratt et al. 2020; Vitacco et al. 2008). Although a recent study found a small, positive relationship between some aspects of primary psychopathy and cognitive, but not emotional intelligence in a student sample (Watts et al. 2016), the results need replication in order to reach a stronger conclusion about intelligence and psychopathy sub-types.

Thus, at present, we cannot determine whether the success of individuals high in primary psychopathy is based on *actual*, measurable intelligence, or on creating the impression that one is an intelligent person. The career success associated with primary psychopathy suggests individuals high in this trait should be perceived as intelligent (see also O’Boyle et al. 2013 for the *‘evil genius’* idea). The criminality, substance abuse, and impulsivity typical of secondary psychopathy could, in turn, lead to perceptions of lower intelligence. The limited studies which have researched perceptions of intelligence have not looked at the two sub-facets of psychopathy. For instance, Edens et al. (2013) found in a court setting, jury members who perceived murderers in case vignettes as psychopathic also perceived them as intelligent. In another vignette study, it was discovered that lay people perceived psychopaths as having superior intelligence (Furnham et al. 2009), whilst those who are perceived as intelligent are more likely to receive more lenient sentences (Brown et al. 2008). Contradictory evidence exists in the form of face-to-face interactions between participants, where high-psychopathy individuals are not rated as particularly high or low in intelligence (Rauthmann 2012). There is a gap in the research in looking at perceptions of intelligence of individuals high in primary and secondary psychopathy, something we will address in the present study.

In this research, we will focus not just on perceptions of intelligence, but also how the participant’s own psychopathy influences this process. Overall, individuals high in psychopathy tend to have a negative heuristic of others (Black et al. 2014), which would suggest that they would rate others as being less intelligent. However, previous research has not looked at how psychopathy affects the perceptions of others who are high or low in psychopathy. In the domain of mate choice, there is some evidence for ‘likes attract’, suggesting high-primary and high-secondary psychopathy relates to favourable perceptions of similar individuals as potential partners, especially in women (Blanchard et al. 2016). As women are more attracted to those who they perceive as intelligent (Karbowski et al. 2016), it would be expected that psychopathy for women has an association with perceptions of intelligence in high psychopathy opposite-sex individuals.

Evaluation of others is often clouded by factors such as intra-sexual competition for mates (Agthe et al. 2010). Individuals may perceive same-sex individuals negatively due to a sexual attribution bias (Agthe et al. 2008) and so consequently, in this study, we only look at the perceptions of intelligence for the opposite sex.

In summary, the present research will investigate the perceptions of intelligence of opposite-sex individuals, using vignettes which describe people who are high or low in primary or secondary psychopathy. We hypothesise that primary-psychopathy vignettes are perceived as more intelligent than secondary-psychopathy vignettes, and that individuals who are high on the particular sub-type themselves perceive the same high-psychopathy sub-type vignettes as being more intelligent. To the best of our knowledge, this is the first study to investigate the perceived intelligence of the two psychopathy sub-types.

## 2. Materials and Methods

### 2.1. Participants and Procedure

Ethical approvals were sought and granted by the University of Liverpool’s Institute of Psychology, Health, and Society Research Ethics Committee [ref:- IPHS-1415-017]. Four hundred and fifty-eight participants (384 females; *M*_Age_ = 24.74, *SD*_Age_ = 10.83) took part in an online study entitled: “Environment, Psychopathology, and Mate Selection”. The online survey was rolled out through a university sample and community sample simultaneously, meaning participants were drawn from a global English-speaking population, with a majority subset based at the host university. The survey took on average 20 min to complete. Thirty-eight participants who identified as homosexual or bisexual were removed from the final dataset, due to being underpowered for any meaningful sub-group analysis. Therefore, all participants included in this analysis identified as heterosexual and therefore rated opposite-sex vignettes. The survey was advertised to students at a university in the North-West of England (in exchange for course credit) and via snowballing to participants through social media and online study participation websites. The first page of the study stated a brief outline and ethical considerations of the study, which was followed by ratings of twelve character profile vignettes on a range of items (not all reported here), with the final section of the study asking each participant to complete a self-report scale of psychopathy.

### 2.2. Measures

We created twelve profiles (three high-primary psychopathic; three low-primary psychopathic; three high-secondary psychopathic; three low-secondary psychopathic), using traits from the Manual for the Self-Report Psychopathy (SRP-III) Scale (Paulhus et al., forthcoming), using aspects of the scale to develop profiles and histories for each vignette character. Vignettes were similar in length and in number of descriptions for each fictional person and met Gould’s (1996) check-list points for validity and reliability when developing vignettes for research studies. For female participants, fictitious male names were used. The same vignettes were used for male participants, except the names and pronouns were changed to be female (see Appendix A for actual vignettes used). Participants were asked to rate these 12 vignettes on the likelihood of the statement being perceived as true, on two proxies for intelligence: a high IQ and university attendance (as in Deary and Johnson 2010). The way the questions were presented to the participant was as follows: Q. *How likely is it that the person in this vignette*: (1) *…has attended University?* (2) *…has a high IQ?* The possible responses to whether the person described in the vignette had attended university or had a high IQ were presented on an eleven-point Likert scale from which they could select the following: *0 = extremely unlikely, 5 = neither likely nor unlikely, 10 = extremely likely*. As the perceptions for high IQ and university attendance were significantly positively correlated (all *r*’s 0.53–0.71, and *p*’s < .001), we combined the two together to form an overall index of perceived intelligence.

All participants then completed the 26-item Levenson Self-Report Psychopathy Scale (LSRP; Levenson et al. 1995; validated by Lynam et al. 1999). Participants responded on how much they agreed (1 = Disagree Strongly; 4 = Agree Strongly) with statements relating to primary psychopathy (of which there were 16 items) and to secondary psychopathy (a further 10 items). Scores were summed in accordance with guidance from Brinkley et al. (2001) to give each participant two scores for each psychopathy sub-type (primary and secondary), which were used for analyses.

We decided on the use of different psychopathy scales for vignette development and self-reported psychopathy to prevent circular responses of participants rating themselves in relation to the vignettes.

### 2.3. Data Analysis

Data were cleaned and analysed using R. To analyse data in respect of the hypotheses, an ANCOVA was administered to account for the participants’ own psychopathy.

## 3. Results

### 3.1. Descriptive Statistics

In Table 1, we present the descriptive statistics of the complete sample, and the sub-samples of student and community participants. To assess for group differences, independent *t*-tests were administered. There were no significant differences between student or community samples or the percentage of females in each respective sample (t(456) = 1.09, *p* = .28). The community sample had a significantly (t(456) = 15.52, *p* < .001) higher mean age (M = 35.69, SD = 13.64) than the student sample (M = 20.98, SD = 6.24). There was a significant association between the samples and country of origin of participants (t(456) = 54.32, *p* < .001), whereby the student sample had significantly more individuals from the UK and less from other countries than the community sample.

### 3.2. Main Analysis

To investigate whether the perceptions of intelligence vary as a function of vignette psychopathy level (high; low), sub-type (primary; secondary), individual psychopathy level (high; mixed; low), and gender of participant (male; female), we conducted a mixed 2 × 2 × 2 ANCOVA. Repeated measures factors included vignette psychopathy level, psychopathy sub-type, and between-subject factors of participant gender. Individual’s primary and secondary psychopathy levels were the covariate.

There was no significant interactions or main effect of gender on perceived intelligence (F(1, 437) = 2.236, η^2^_p_ = 0.002, *p* = .136). There was a main effect of psychopathy level (F(1, 437) = 34.552, η^2^_p_ = 0.029, *p* < .001), but the effect size was small to moderate. High-psychopathy vignettes were rated as significantly less intelligent than low-psychopathy vignettes (t(1, 437) = −16.689, *p* < .001). There was no significant main effect of subtype on perceived intelligence (F(1, 437) = 2.070, η^2^_p_ = 4.410 × 10^−4^, *p* = .151). Psychopathy and sub-type had a significant interaction on perceived intelligence (F(1, 437) = 4.876, η^2^_p_ = 0.002, *p* < .05), but this had a small effect size. Bonferroni-corrected *post-hoc* analyses showed that high-secondary psychopathy vignettes were rated as significantly less intelligent than high primary (t(1, 437) = −24.949, *p* < .001), low primary (t(1, 437) = −22.318, *p* < .001), and low secondary (t(1, 437) = −7.490, *p* < .001). Furthermore, high-primary psychopathy vignettes were rated as significantly less intelligent than low primary (t(1,4 37) = −4.128, *p* < .001) and low secondary (t(1, 437) = −7.490, *p* < .001). Low-secondary psychopathy vignettes were rated as significantly more intelligent than low-primary vignettes (t(1, 437) = 4.354, *p* < .001). There was a significant interaction between the sub-type of the vignette and the individual’s own primary psychopathy score (F(1, 437) = 8.733, *p* < .01, η^2^_p_ = .020).

We also correlated the participant psychopathy scores and their ratings of intelligence of high and low primary and secondary psychopathy vignettes (see Table 2). As individuals’ psychopathy scores increased—irrespective of type—there was a significant positive correlation with perception of high-primary psychopathy vignette intelligence. Individuals’ primary psychopathy score was positively correlated with the perception of intelligence of low-secondary psychopathy vignettes.

In Table 2, we present the means and standard deviations for perceived intelligence of the vignettes and correlations with between participant and vignette psychopathy. Table 2 also contains the Pearson’s correlation co-efficient between the participants own psychopathy scores and their perceptions of intelligence in the vignettes.

## 4. Discussion

Previous research has documented the association between primary psychopathy and enhanced academic and career success, and likewise the inverse relationship for secondary psychopathy and success (Akhtar et al. 2013; Gao and Raine 2010; Hassall et al. 2015; Howe et al. 2014; Ullrich et al. 2008). Our study indicates that perceptions of intelligence for psychopathy sub-types mimic this same pattern—regardless of the sex of the participant. Regarding intelligence, high-secondary psychopathy vignettes were perceived as significantly lower and high-primary psychopathy vignettes as significantly higher. Interestingly, low-primary and low-secondary psychopathy vignettes were also perceived as having high intelligence. This suggests people may be using both high *and* low empathy, as well as low impulsivity, as a proxy for intelligence. In contrast, high impulsivity may be used as a proxy for lower intelligence (see Loeber et al. 2012, for a study on impulsivity-IQ link).

It is possible that perception of intelligence is related to the public presentation of self (e.g., self-control in social settings; Uziel 2010). It was found by Murphy (2007) that people were attributed greater intelligence if they were good at impression management, and likewise positive impression management is uncharacteristic of high-secondary (but not high primary) psychopathy sub-facets (Gillard and Rogers 2015). It would therefore be interesting to further examine whether perceptions of self-control are the driving force behind viewing high-secondary psychopathy vignettes as being less intelligent.

We found a difference between perceptions of high- and low-primary psychopathy vignettes, implying that descriptions of both high and low callous, exploitative individuals are not perceived as equally intelligent. Whilst interesting, this is unsurprising, as the literature already suggests those exhibiting high-primary psychopathy may be seen as somewhat emotionally deficient, but not disturbed (Yildirim and Derksen 2015), are also not seen to be risky decision makers (Dean et al. 2013), and can therefore assimilate into normal society, mirroring the normative behaviours of their counterparts who are low in psychopathy. Though this may be the case, the differences between people who display high- and low-primary psychopathy are notable enough to be assessed differently in this study, and so are perhaps not as subtle as once thought.

Further literature which may help to explain this phenomenon could be seated within leadership and management. High-psychopathy individuals are motivated by status and power and may seek leadership positions in corporations (Babiak et al. 2010). This may be easier to achieve if those individuals are perceived by others as being intelligent. Mathieu et al. (2015) report psychopathy itself may be synonymous with emergent leaders and psychopathic traits are tantamount to leadership identity. Contrary to this, high emotional intelligence and empathy (characteristics of low-primary psychopathy) have also been identified as qualities which can lead to effective leadership (Kellett et al. 2002). The disparity may be reconciled by suggesting multiple routes to successful leadership. In sectors which require a cool-headed, authoritative, rational decision-maker, high-primary psychopathy characteristics may be deemed more appropriate in a leader, as has been noted in state leaders from across the globe (Lilienfeld et al. 2012; Steinberg 2008). However, where decision-making requires a nurturing approach, based on emotional intelligence, low-primary psychopathy characteristics in leaders are more desirable; this has most prominently been identified in traditionally care-centred occupations instead of the business sector (Eason 2009; Holmes et al. 2003; Ilić 2008). Both high- and low-primary psychopathic leadership styles are deemed to be necessary in their given contexts, and each leader—despite level of primary psychopathy—is perceived as intelligent by others and is looked-up to for guidance and strategic direction. More work is required to fully understand what may be fundamentally the same between people scoring high and low in primary psychopathy for them to achieve similar ratings of perceived intelligence by those who rate them. To aid this, we suggest repeating experiments of this study’s nature, but including other personality questionnaires, which may provide more rounded profiles of participants. Notably, we would recommend the use of the ‘Big Five’ personality traits, with a focus on the NEO facet (McCrae et al. 2005) to better understand the relationship of psychopathological traits of neuroticism, extraversion, and tendencies of openness with both psychopathy and intelligence perceptions.

Interestingly, we also found evidence for the ‘likes attract’ idea, indicating those who themselves score highly in primary psychopathy may perceive primary psychopathy individuals as more intelligent. The ‘negative other’ heuristic typical to individuals high in psychopathy (see Black et al. 2014) seems to be lifted when assessing others who are similar to the self. To our knowledge, few studies have looked at the psychopathy sub-types and inter-personal perceptions for individuals who have similar personality traits. A study on mate choice found an effect on primary psychopathy and preference for high-primary psychopathy vignettes as romantic partners, especially in women (Blanchard et al. 2016). It is possible that mate preference operates via increased perceptions of intelligence. We also found other positive correlations, albeit of smaller magnitude. For example, women high in secondary psychopathy viewed high-primary psychopathy vignettes as intelligent, and women high in primary psychopathy rated low-secondary psychopathy vignettes as intelligent. There are not many studies investigating how psychopathy relates to how individuals perceive others (although see Mahaffey and Marcus 2006) and future studies should investigate this further in different contexts (e.g., mate choice, and the corporate world).

The current study is not without its limitations. First, we had a relatively small number of men, as compared with women in the study, and the results for the male sample should be treated with the appropriate caution. Second, participants may have demonstrated the ‘halo effect’, whereby because participants were being asked to rate the intelligence of characters from psychopathic vignettes and score their own psychopathy, they may have altered their scoring (i.e., they may have simply been substituting how ‘good’ or similar to themselves each vignette was, as a proxy for perceived intelligence). However, asking participants to score their own psychopathy after they had rated the vignettes should have reduced the effect of this issue. Additionally, as the online study was advertised to students and the researchers’ social networks, the survey can be assumed to capture only a small part of a predominantly middle-class population. Research has found socio-economic status, for example, is a powerful predictor of how we perceive others (Hall et al. 2015), and our results may not generalise to those of the very lowest socio-economic status, or those of very great wealth. Third, the online nature of the study means we had very little control over our participants. Despite this, online studies have recently gained popularity within psychological research and prove to be a reliable means to test participants (Hewson 2014). We would, however, suggest laboratory replications, possibly using vignettes combined with facial morphs of high and low primary and secondary psychopathy (as in Lyons and Simeonov 2016) to see whether the context of written information is different to written and visual information. It would also be interesting to see how primary- and secondary-psychopathic individuals are rated by others during interactions in a laboratory environment, and whether the dominant features of primary psychopathy (Manson et al. 2014) also relate to higher perceived intelligence. Linking to the format of this study, the vignettes themselves could be open to interpretation by participants (as cautioned by O’Dell et al. 2012). To counter this, we followed previously employed practices of vignette development to avoid such limitations (see Hughes and Huby 2004). In doing so, we also made sure to meet the requirements for designing vignettes as set out by Gould (1996), whereby internal reliability was achieved (by referring to the existing literature of people high and low in primary and secondary psychopathy and their associated life outcomes) and by developing these vignettes in line with a well-used scale (the SRP-III by Paulhus et al., forthcoming). We therefore deem the vignettes used in this study to be both valid and reliable for empirical testing and would suggest following these same protocols in future vignette studies which may aim replicate or add to our findings. To expand on this research, future studies may wish to explore the social intelligence of participants with high levels of psychopathy as well as their ability to leverage their understanding of the motivations of others. Future research may also want to take a full socio-demographic, psychiatric, and criminal history as well as a score of intelligence from participants to add context and richness to study findings.

## 5. Conclusions

In summary, intelligence is often at the forefront of human observation, and intelligence can open or close doors, rendering it crucial to accomplishment in the workplace. For example, those who are rated as intelligent may be more likely to get promotions and less likely to be made redundant. Indeed, the link between primary psychopathy and career success could potentially be moderated by the perceptions of intelligence, something which should be investigated in future research. In the legal setting, those who are rated as intelligent receive more lenient sentences. This indicates individuals high in primary psychopathy could receive shorter sentences than those high in secondary psychopathy. Our findings further the understanding on why primary psychopathy is identified as the successful and secondary psychopathy as the unsuccessful facet of the psychopathy construct. Worth noting, of course, is this study’s reliance on heterosexual cross-sex raters of vignettes, meaning there could be an intentionality at play here where intelligence, psychopathy, and the search for a potential mate are coalescing to produce these results. Future research should extend this investigation. It may also therefore be appropriate for the research in this area to re-evaluate psychopathy as no longer standing as a unitary concept, in light of how diverse primary and secondary psychopathy appear in presentation, in outcomes, and now also in how they are perceived intellectually.

## Figures and Tables

**Table 1:** Sample characteristics.

	Student n = 339	Community n = 119	Total n = 458
**Mean Age (SD)**	20.98 (6.24)	35.69 (13.64)	24.74 (10.83)
**Percentage Female**	84.96	80.67	83.85
**Country of Origin %**			
United Kingdom	82.4	54.1	75.04
North America	5.6	20.1	9.37
Europe	5.4	16.7	8.34
Asia	4.8	2.7	4.25
Other	1.8	6.4	3.00

**Table 2:** Mean and SD of intelligence rating of vignettes, and correlations between vignette psychopathy and rater.

	Mean (SD)	Primary Psychopathy	Secondary Psychopathy
High Primary Vignette	5.53 (1.59)	0.26 **	0.11 *
Low Primary Vignette	6.20 (1.21)	0.02	0.01
High Secondary Vignette	3.09 (1.49)	−0.011	0.05
Low Secondary Vignette	6.68 (1.34)	0.12 *	−0.05

* *p* < .05 ** *p* < .001.

## Data Availability

Materials associated with this study can be found in the Appendix A. Data is not stored in a research repository and therefore is not accessible.

## Appendix A. Character Profile Vignettes

### Appendix A.1. High Primary Psychopathy

Meet Jenny/John, s/he describes her/himself as: A tough-minded individual, who finds it easy to manipulate people. S/he is not really an emotional person, and rarely cries at movies, even when the films are really sad. S/he thinks that overall, people are far too sensitive, and should toughen up a little.

Meet Paige/Paul, s/he describes her/himself as: An individual who can easily step on others to get what s/he wants. S/he can talk other people into doing what s/he wants them to do, and thinks that people can be easily fooled. S/he thinks that s/he is good at concealing a lie, and could easily beat a lie-detector.

Meet Danielle/David, s/he describes her/himself as: A cold-hearted individual, who has no time to waste on family or friends that don’t offer any benefits. S/he often uses flattery to coax people to her/his way of thinking and seeks out her/his thrills by often assuming another persona, taking part in a big scam or watching horror films.

### Appendix A.2. Low Primary Psychopathy

Meet Rosie/Richard, s/he describes her/himself as: Somebody who has a soft heart, and feels really sorry when s/he sees other people or animals suffering. S/he would never take advantage of other people, and thinks that people can usually tell if s/he’s lying.

Meet Lisa/Luke, s/he describes her/himself as: A person who has a strong aversion to violence and dislikes the thought of violent sports and movies. Likewise, s/he would never go out of her/his way to make anyone upset and feels very guilty if s/he offends someone, even when not intentionally.

Meet Sara/Stephen, s/he describes her/himself as: An honest person in touch with their emotions, who believes that most people can be trusted to be honest too. S/he is always truthful and doesn’t pretend to people in order to achieve something, thinking, because of this they won’t pretend to her/him either.

### Appendix A.3. High Secondary Psychopathy

Meet Nancy/Nick, s/he describes her/himself as: Somebody who enjoys taking risks, whether it is gambling, taking drugs, driving at high speed, or engaging in violent sports. S/he is an impulsive person, who lives life here and now rather than worries about the future.

Meet Hollie/Henry, s/he describes her/himself as: A reckless individual who rebels against any authority often getting in trouble with the law with her/his gang when s/he was younger. Since then s/he has been arrested for attempting to run someone over and associates closely with people who have served prison sentences.

Meet Carla/Chris, s/he describes her/himself as: Somebody without a plan, preferring to live dangerously, sometimes just for the thrill it gives her/him. Every now and again, s/he is known to carry a weapon for protection, and has a history of threatening people for valuables, shoplifting and also motor theft.

### Appendix A.4. Low Secondary Psychopathy

Meet Monica/Matthew, s/he describes her/himself as: A sensitive person who would never do dangerous things just for the thrill of it. S/he likes to plan out his weekly activities, and never misses appointments. S/he hates high speed driving, and never gets in fights with others.

Meet Gabby/George, s/he describes her/himself as: Somebody who is never bored and with a life plan. As a rule follower, s/he has never taken part in illegal activities, and prefers an office job over something that offers any danger or insecurity.

Meet Olivia/Oscar, s/he describes her/himself as: An individual who is very rarely impulsive, and so has never taken hard-drugs and doesn’t enjoy gambling for real money. S/he prefers to get to know people before s/he engages in a relationship with them and has never been involved in any trickery for money.
